# Abundant Development of Agaricales Fungi on Livingston Island, Antarctica: Potential Connections to Climate Change

**DOI:** 10.1007/s00284-026-04947-6

**Published:** 2026-06-09

**Authors:** Fernando Augusto Bertazzo-Silva, Flavia Helena Aires Sousa, Kamille Rodrigues Ferraz, Jorge Renato Pinheiro Velloso, Evelise Leis Carvalho, Marcos André Pinheiro Velloso, Vanessa dos Anjos Baptista, Adriano Luis Schünemann, Carlos Ernesto Gonçalves Reynaud Schaefer, Jair Putzke

**Affiliations:** 1 Laboratório de Taxonomia de Fungos, Universidade Federal do Pampa (UNIPAMPA), Rua Aluízio Barros Macedo, s/n. BR 290 – km 423, São Gabriel, Rio Grande do Sul 97307-020 Brazil; 2 Faculdade do Centro Educacional Santa Isabel – FACESI, Estrada da Branquinha, 299 – Viamópolis, Viamão, Rio Grande do Sul 94455-000 Brazil; 3 Programa de Pós Graduação em Ciências Biológicas (PPGCB), Universidade Federal do Pampa (UNIPAMPA), Rua Aluízio Barros Macedo, s/n. BR 290 – km 423, São Gabriel, Rio Grande do Sul 97307-020 Brazil; 4 Departamento de Solos, Universidade Federal de Viçosa (UFV), Av P.H. Rolfs, s/n, Centro, Viçosa, Minas Gerais 36571-000 Brazil

## Abstract

Rapid warming across Antarctica is reshaping terrestrial ecosystems, yet the dynamics of macroscopic fungi in this region remain largely undocumented. Here, we present the first ecological and statistical assessment of Agaricales fungi on Byers Peninsula, Livingston Island (South Shetland Islands, Antarctica), based on surveys conducted in February 2023. Across 20 sites, we recorded 2,566 basidiomes representing eight morphospecies in three genera (*Arrhenia, Galerina, and Omphalina*). Community diversity was moderate (Shannon–Wiener H′ = 1.97; Simpson’s D = 0.85), with assemblages dominated by *Omphalina* (68.4% of basidiomes), while *Arrhenia* sp. 1 displayed the broadest spatial distribution (65% of sites). Spatial analyses revealed significant variation in Agaricales abundance among sites (ANOVA, p = 0.016), while multivariate ordination (NMDS based on Bray–Curtis dissimilarity) showed substantial overlap in morphospecies composition across dominant vegetation types, with no significant differences detected by PERMANOVA (p = 0.527). The emergence of diverse basidiome assemblages in such an extreme environment underscores the ecological relevance of fungi in polar ecosystems and highlights their potential as sensitive bioindicators of climate-driven change. This quantitative baseline provides a critical reference for long-term monitoring and advances understanding of fungal contributions to biogeochemical processes in a rapidly changing Antarctic landscape.

**Supplementary Information:**

The online version contains supplementary material available at 10.1007/s00284-026-04947-6.

## Introduction

The 2023 Intergovernmental Panel on Climate Change (IPCC) report underscored an alarming reality: between 2011 and 2020, the global mean surface temperature rose by approximately 0.99 °C relative to the 1850–1900 baseline. This represents the most rapid warming trend since 1970 and an unprecedented pace over the last two millennia [20]. The Antarctic region is not exempt from these changes. The record-breaking heatwave of March 2022, when temperature anomalies reached up to + 39 °C above climatological norms, exemplifies the growing frequency and intensity of extreme warming events in this polar environment [9]. Such events highlight the ecological vulnerability of Antarctica, particularly the Peninsula, which is among the most climate-sensitive areas on the continent [18, 23, 29].

Although Antarctica remains relatively isolated and less impacted by direct human activities, it is no longer biologically insulated from global processes. Rising temperatures facilitate the establishment of non-native species, with the potential to alter ecosystem structure and function even in the absence of deliberate human introduction [17]. One striking manifestation of these changes is the emergence of macroscopic fungi in habitats previously constrained by extreme cold. Fossil evidence indicates that fungi have a long evolutionary history on the continent, dating back to the Permian, yet contemporary records of their development remain scarce [19, 26, 40].

Among these organisms, Basidiomycota fungi are particularly rare, and reports of their reproductive structures (basidiomata) are limited [4, 33]. To date, 21 Agaricales species forming basidiomata have been documented on the continent, mostly in the Maritime Antarctic and occasionally on the western Antarctic Peninsula, usually associated with dense moss mats and vascular plants [8]. Despite their rarity, these fungi play critical ecological roles in nutrient-poor polar ecosystems, contributing to decomposition, nutrient cycling, and symbiotic associations that shape biogeochemical processes [5, 37]. Their scarcity in Antarctica has been linked to both environmental constraints and the absence of woody vegetation and large terrestrial fauna, which in other ecosystems provide important substrates and dispersal pathways [10, 24].

Given their ecological importance and sensitivity to environmental conditions, basidiomycetous fungi represent valuable indicators of ecosystem responses to ongoing climate change. Although mushrooms remain rare, recent trends toward warmer and more humid summers may be creating more favorable conditions for their establishment and visibility [7, 26].

In this context, the present study investigates the distribution, diversity, and abundance of Agaricales fungi on an island of the Antarctic Maritime during February 2023. By documenting basidiomata occurrence and exploring potential environmental drivers, it establishes a baseline for long-term monitoring and advances understanding of the ecological role of fungi in Antarctic terrestrial ecosystems under ongoing climate change.

## Materials and Methods

### Study Area, Sampling, and Species Classification

**Fig. 1:**
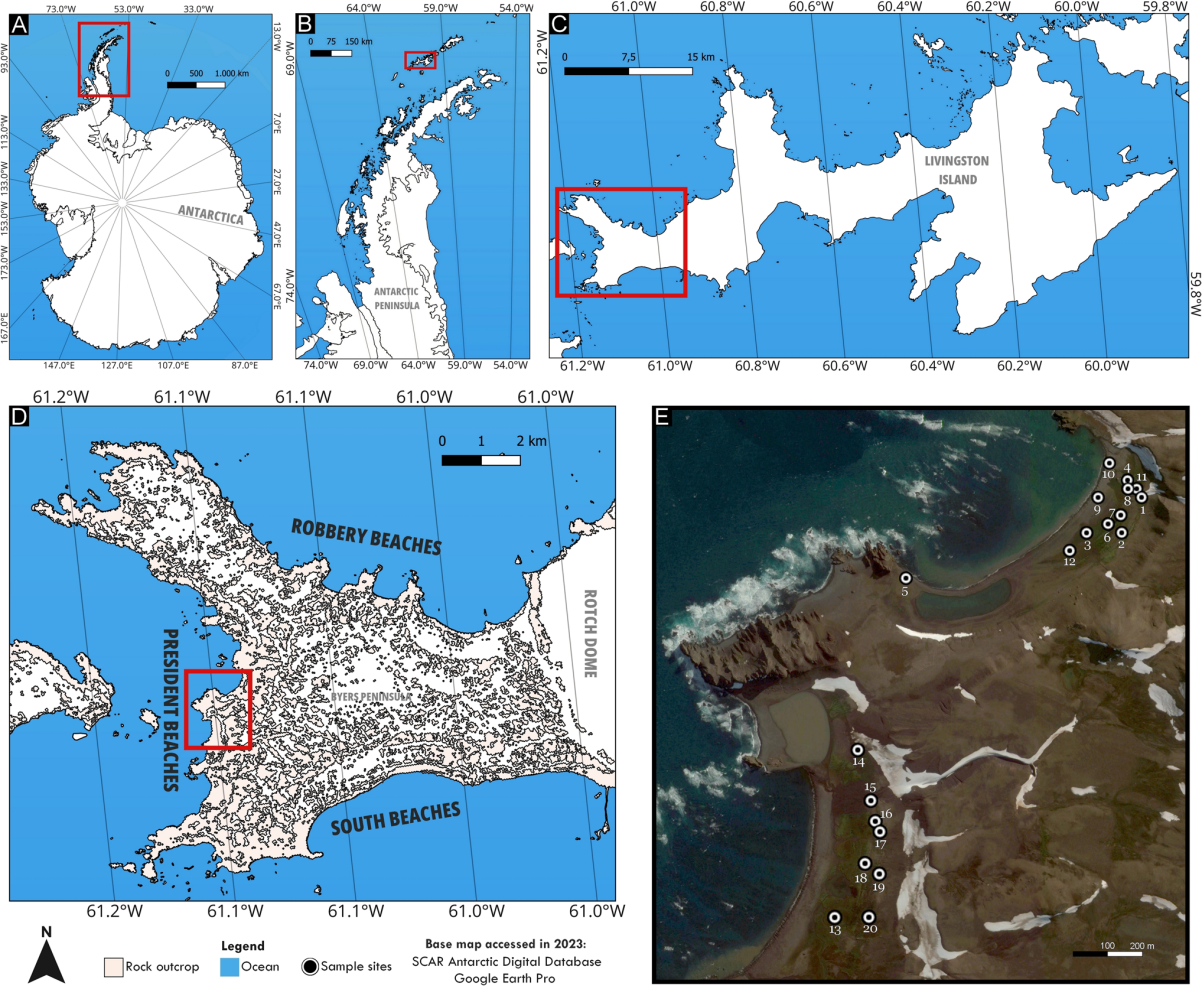
Location map of the collection area. **A** Antarctica. **B** Antarctic Peninsula. **C** Livingston Island. **D** Byers Peninsula. **E** Laager Point with 20 collected points. Created in QGIS 3.32.0 software and designed in PhotoFiltre Studio X 10.12.1 software

Fieldwork was conducted in February 2023 during the XLI Brazilian Antarctic Operation at two coastal sites along President Beaches, Byers Peninsula, Livingston Island, South Shetland Archipelago, Antarctica (Fig. 1). At these locations, twenty sites were randomly selected for data and sample collection. Vegetation composition was documented, and geographic coordinates were recorded at each site. Based on these records, sites were subsequently classified according to their dominant vegetation communities (Table 1).

**Table 1:** Sites selected for data acquisition, sample collection, along with geographic coordinates, and dominant vegetation composition

Sample sites		
Site	Location	Predominant vegetation composition
1	62° 38′ 01″ S 61° 07′ 18″ W	*Sanionia uncinata* (Hedw.) Loeske
2	62° 38′ 05″ S 61° 07′ 23″ W	*Sanionia uncinata*
3	62° 38′ 05″ S 61° 07′ 31″ W	*Deschampsia antarctica* Desv
4	62° 37′ 59″ S 61° 07′ 21″ W	*Warnstorfia sarmentosa* (Wahlenb.) Hedenäs
5	62° 38′ 10″ S 61° 08′ 12″ W	*Deschampsia antarctica*
6	62° 38′ 04″ S 61° 07′ 26″ W	*Sanionia uncinata*
7	62° 38′ 03″ S 61° 07′ 23″ W	*Sanionia uncinata and Deschampsia antarctica*
8	62° 38′ 00″ S 61° 07′ 21″ W	*Sanionia uncinata and Bryum* sp.
9	62° 38′ 01″ S 61° 07′ 28″ W	*Deschampsia antarctica and diverse mosses*
10	62° 37′ 57″ S 61° 07′ 25″ W	*Deschampsia antarctica*
11	62° 38′ 00″ S 61° 07′ 19″ W	*Warnstorfia sarmentosa*
12	62° 38′ 07″ S 61° 07′ 35″ W	*Deschampsia antarctica*
13	62° 38′ 44″ S 61° 08′ 26″ W	*Warnstorfia sarmentosa*
14	62° 38′ 28″ S 61° 08′ 22″ W	*Sanionia uncinata*
15	62° 38′ 33″ S 61° 08′ 19″ W	*Sanionia uncinata and Deschampsia antarctica*
16	62° 38′ 35″ S 61° 08′ 18″ W	*Warnstorfia sarmentosa*
17	62° 38′ 36″ S 61° 08′ 17″ W	*Sanionia uncinata*
18	62° 38′ 39″ S 61° 08′ 20″ W	*Warnstorfia sarmentosa*
19	62° 38′ 40″ S 61° 08′ 17″ W	*Sanionia uncinata*
20	62° 38′ 44″ S 61° 08′ 19″ W	*Warnstorfia sarmentosa*

Fungal occurrence was assessed using the walking method, a standard approach in Agaricales surveys [27]. As basidiomes were encountered, their abundance was recorded and specimens were provisionally identified in the field based on macroscopic characters. Representative basidiomes of each morphospecies were collected, dried, transported to Brazil, and subsequently deposited in the Bruno Edgar Irgang Herbarium under accession numbers HBEI 133, HBEI 134, HBEI 135, HBEI 136, HBEI 143, HBEI 144, HBEI 145, and HBEI 146.

Given the ecological scope of this study, which focused on abundance patterns and spatial distribution, molecular identification of all recorded basidiomes was neither feasible nor essential. Instead, basidiomes were documented and grouped into morphospecies based on shared morphological characters, with identifications restricted to the genus level, an accuracy attainable in the field without molecular tools. This conservative approach minimizes taxonomic overestimation and ensures that the reported patterns reflect the observed assemblages rather than assumed species-level identities.

### Data Analysis and Statistics

All statistical analyses were performed in R [28] using the vegan, ggplot2, and tidyverse packages [25, 38, 39].

Sampling adequacy and total species richness were first evaluated using species accumulation curves generated from all sampling sites. To estimate undetected diversity and account for the influence of rare taxa, three non-parametric richness estimators were applied: Chao1 [12, 13], Jackknife [11], and bootstrap [14], providing conservative estimates of total community richness.

Fungal alpha diversity was quantified for each site using the Shannon–Wiener (H′) and Simpson’s (1–D) diversity indices [34, 36]. Differences in diversity patterns among the 20 study sites were assessed descriptively. To visualize distributional trends, we generated an abundance plot for the eight identified morphospecies, highlighting their relative frequencies and spatial occurrence.

As an exploratory assessment of overall similarity among sampling sites, patterns of fungal composition and abundance were first examined to obtain a preliminary overview of compositional similarity. Building on this initial assessment, differences in Agaricales abundance among sites were subsequently evaluated using one-way analysis of variance (ANOVA) [16], with F-tests applied to test the null hypothesis of no significant variation in abundance across sampling sites.

To evaluate whether spatial variation in Agaricales abundance was associated with changes in vegetation community composition, multivariate patterns were explored using non-metric multidimensional scaling (NMDS) based on Bray–Curtis dissimilarities derived from morphospecies abundance data across sampling sites. The NMDS ordination was used to visualize similarities and differences in assemblage composition among sites and across dominant vegetation types.

The influence of vegetation type on assemblage composition was formally tested using a permutational multivariate analysis of variance (PERMANOVA) applied to the same Bray–Curtis distance matrix, with significance assessed through 999 permutations.

## Results

**Fig. 2:**
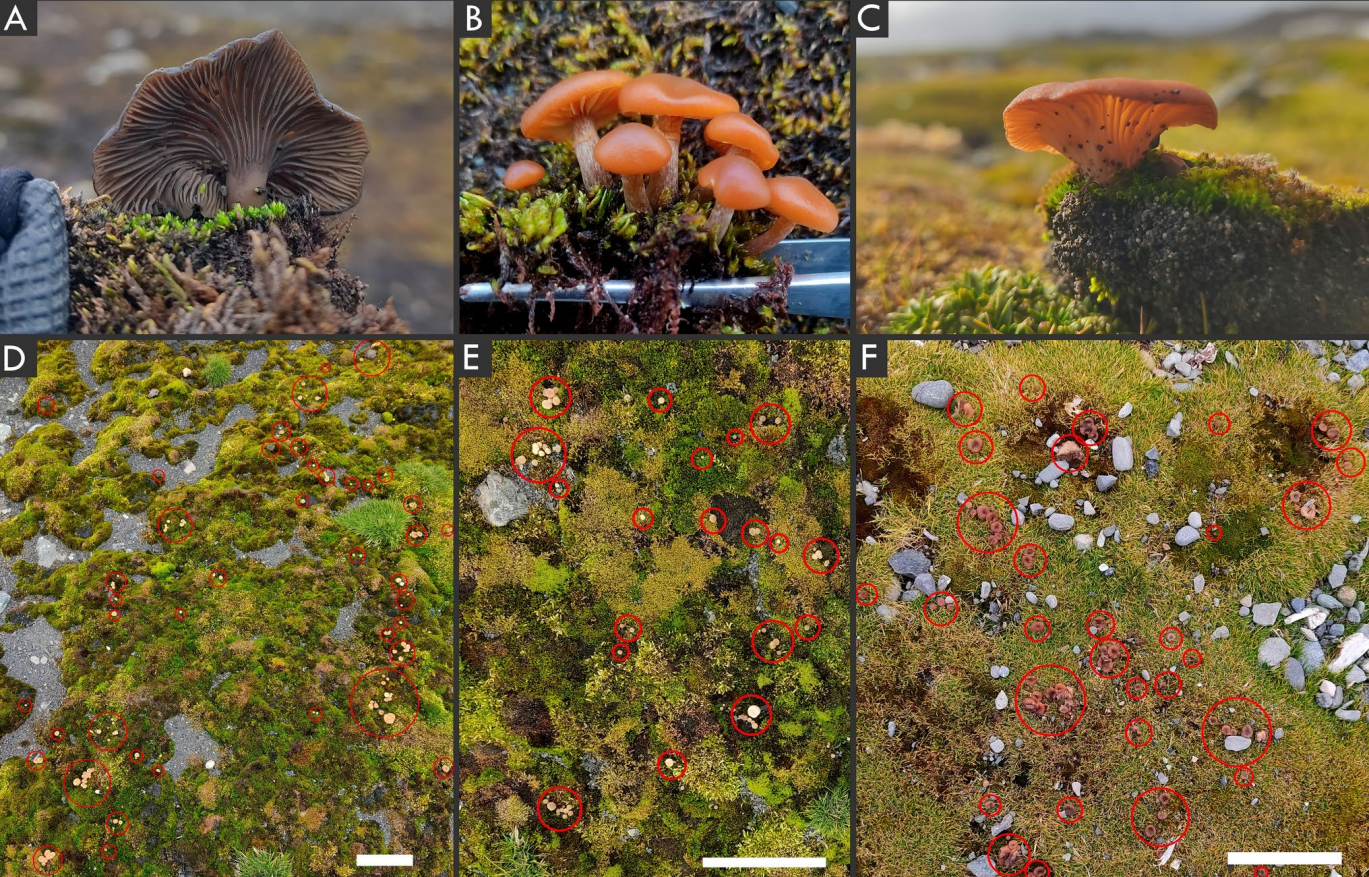
Representative basidiomes and sampling sites documented in this study. **A** Representative specimen of the genus *Arrhenia*. **B** Representative specimen of the genus *Galerina*. **C** Representative specimen of the genus *Omphalina*. **D**–**F** Examples of sampling sites analyzed in this study. Source: Authors, 2023

Field surveys yielded 2,566 basidiomes, representing eight Agaricales morphospecies across three genera (Fig. 2A–C): *Arrhenia* sp. 1 (HBEI 136), *Galerina* sp. 1 (HBEI 135), and six *Omphalina* morphospecies (HBEI 133; HBEI 134; HBEI 143; HBEI 144; HBEI 145; HBEI 146).

**Fig. 3:**
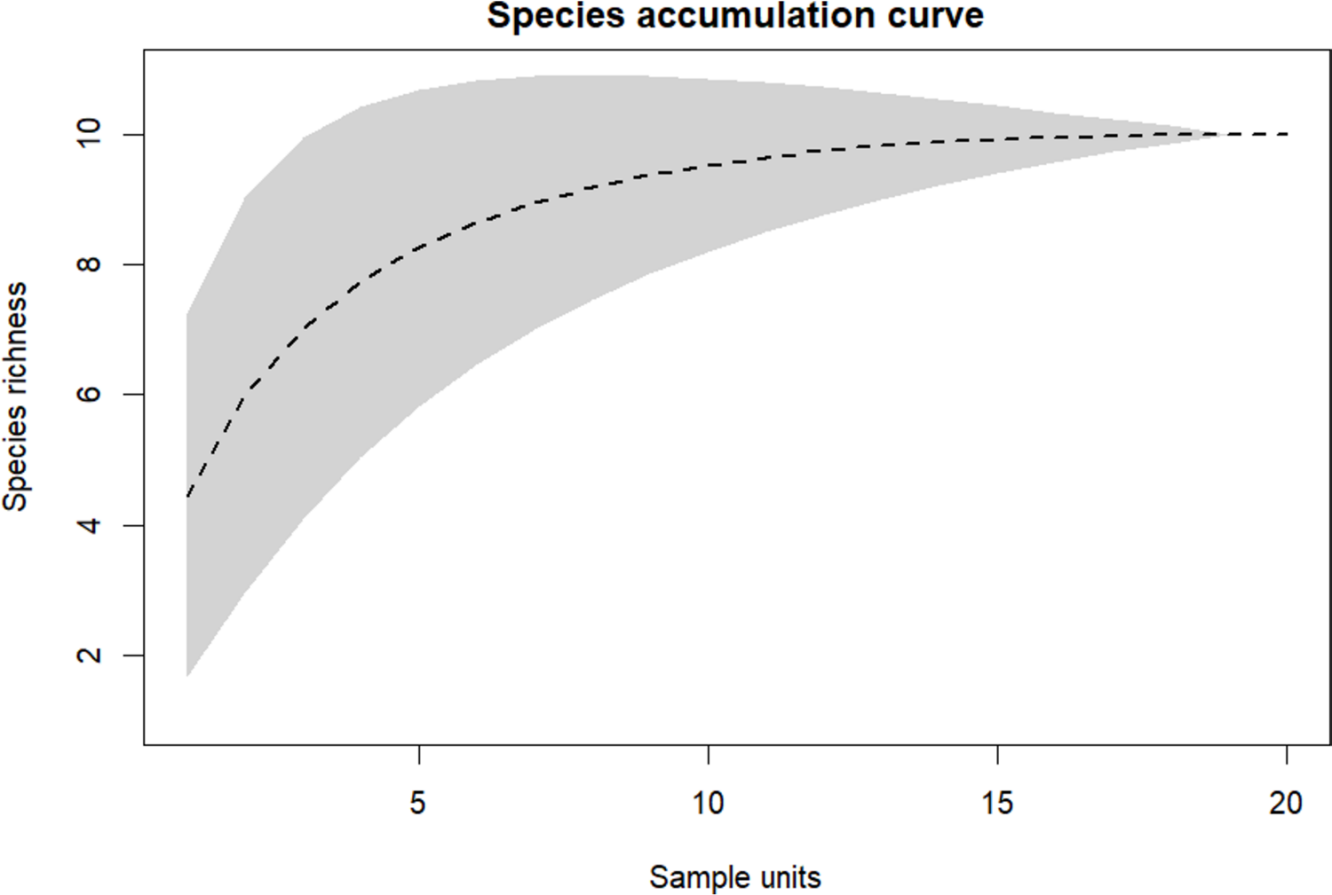
Morphospecies accumulation curve for Agaricales across sampling sites, showing the cumulative number of observed morphospecies in relation to sampling effort. The curve approaches an asymptote around sampling site 15, indicating sufficient sampling to capture most Agaricales morphospecies present in the study area

The morphospecies accumulation curve approached its asymptote near sampling site 15 (Fig. 3), demonstrating that the collection effort sufficiently captured the Agaricales morphospecies diversity in the study area. This stabilization pattern indicates that most resident morphospecies were detected by this stage of sampling. Richness estimators provided consistent validation, with both Chao1 and first-order Jackknife (Jack1) predicting eight morphospecies, matching the observed richness. The second-order Jackknife (Jack2) estimated 7.14 morphospecies, while Bootstrap analysis yielded an estimate of 8.20 morphospecies. The convergence of these independent estimators supports the adequacy of the sampling effort and the reliability of the reported morphospecies richness.

Given the adequate sampling coverage and the consistency among richness estimators, community diversity was moderate (Shannon–Wiener index H′ = 1.97; Simpson’s index D = 0.85), indicating uneven assemblages dominated by a few taxa (Fig. 2D–F).

**Fig. 4:**
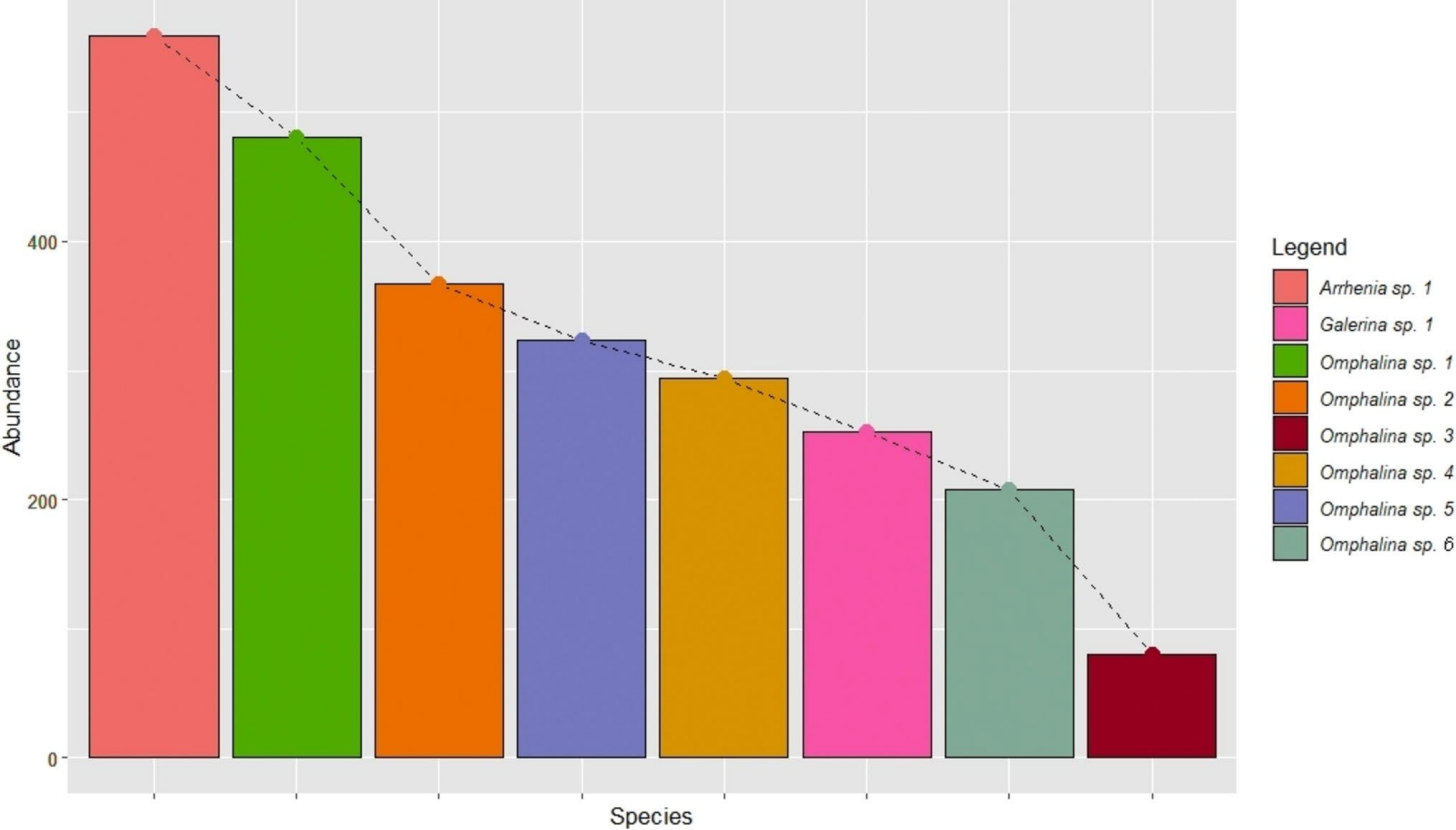
Total abundance of Agaricales morphospecies recorded across all sampling sites. Bars represent the cumulative number of basidiomes per morphospecies observed during the survey, grouped by genus. The dashed line highlights the decreasing trend in morphospecies abundance across the assemblage

Analysis of taxonomic composition and distribution patterns revealed that *Omphalina* was the most abundant genus, accounting for 68.4% of all collected specimens (1,754 basidiomes) and encompassing six morphospecies (Table 2; Fig. 4). In contrast, at the morphospecies level, *Arrhenia* sp. 1 exhibited the highest site frequency, occurring in 65% of sampling locations (13 out of 20 sites) and contributing 559 basidiomes. This discrepancy between genus-level abundance and morphospecies-level distribution reflects distinct ecological patterns, whereby *Omphalina* collectively dominated total basidiome production, while *Arrhenia* sp. 1 showed the broadest spatial occupancy among all recorded morphospecies.

**Table 2:** Species distribution at sample sites.** A1:** *Arrhenia* sp. 1*.*** G1:** *Galerina* sp. 1. **O1:** *Omphalina* sp. 1. **O2:** *Omphalina* sp. 2. **O3:** *Omphalina* sp. 3. **O4:** *Omphalina* sp. 4. **O5:** *Omphalina* sp. 5. **O6:** *Omphalina* sp. 6

SP	Quantification of basidiomes in each research site																			
	1	2	3	4	5	6	7	8	9	10	11	12	13	14	15	16	17	18	19	20
A1	98	95	156	-	-	19	-	46	88	-	-	-	18	13	2	12	4	6	-	2
G1	-	-	-	-	-	-	-	-	-	-	-	-	85	-	-	168	-	-	-	-
O1	109	-	-	37	-	-	106	0	131	-	31	5	-	62	-	-	-	-	-	-
O2	9	1	-	-	-	-	-	2	195	62	-	10	-	88	-	-	-	-	-	-
O3	-	-	-	-	-	-	-	-	-	10	-	-	-	69	1	-	-	-	-	-
O4	-	-	-	-	-	-	-	-	-	-	-	-	-	256	36	-	-	-	2	-
O5	96	-	-	-	-	-	-	-	-	104	36	36	-	52	-	-	-	-	-	-
O6	14	5	-	31	10	62	2	9	-	50	-	25	-	-	-	-	-	-	-	-

**Fig. 5:**
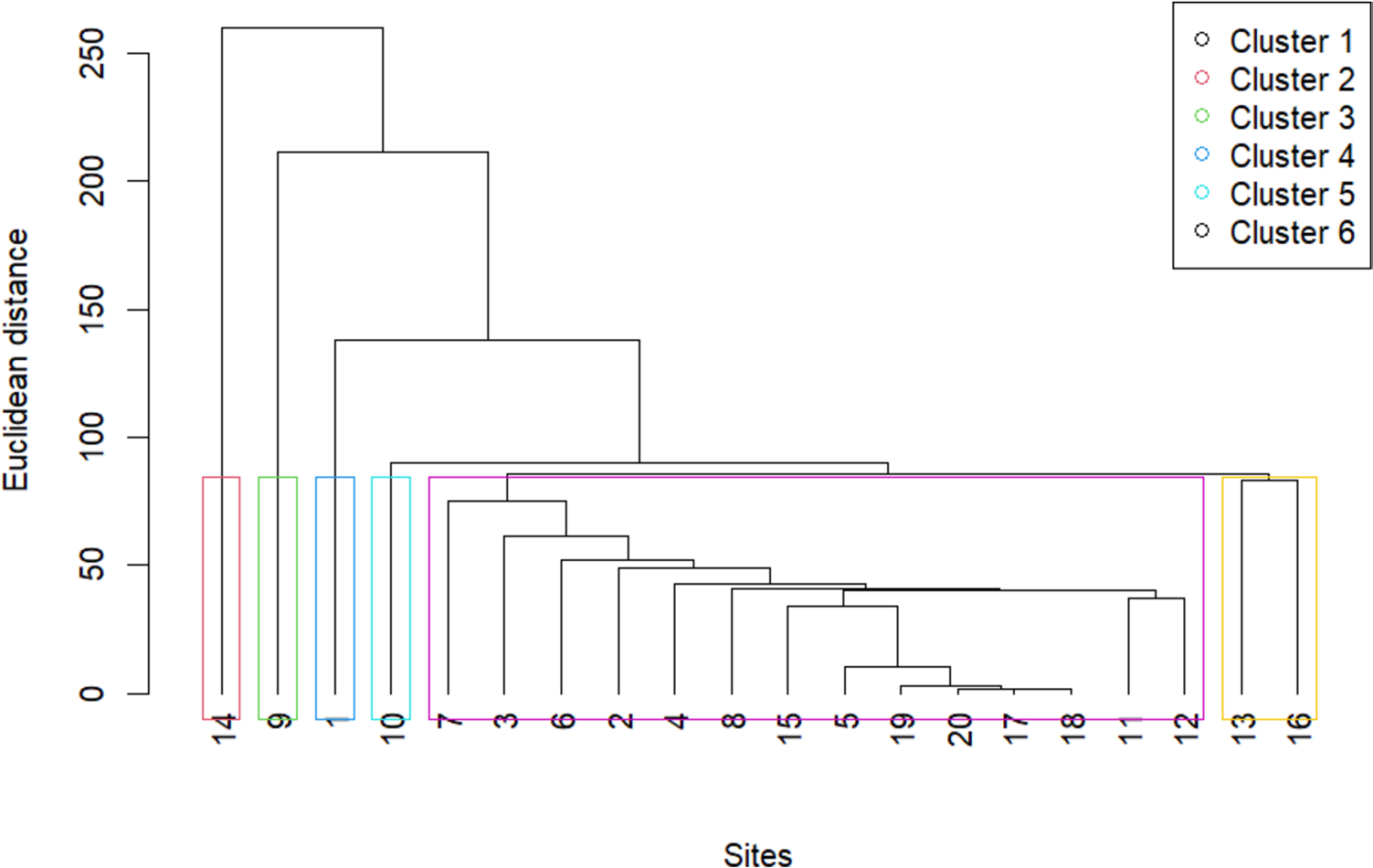
Exploratory visualization of similarity among sampling sites based on Agaricales morphospecies composition and abundance patterns. Sites that cluster more closely share more similar assemblages in terms of morphospecies richness and relative abundance

Exploratory analysis of fungal diversity and occurrence patterns identified three major groupings among sampling sites (Fig. 5). A primary cluster comprised sites 2–8, 10–12, and 15–20, with internal substructure suggesting microenvironmental variation within this broadly similar group. Sites 1, 9, 10, and 14 formed a distinct cluster characterized by unique species composition, while a third, isolated clade included only sites 13 and 16, indicating marked mycological distinctness. Together, these groupings reveal clear biogeographic structuring across the study area, with cluster proximity reflecting similarity in species richness and community composition.

Univariate analysis revealed significant spatial variation in Agaricales abundance among sampling sites (one-way ANOVA, p = 0.016), rejecting the null hypothesis of equal means at α = 0.05. The F-test (p = 0.01604) indicated that at least one site differed significantly in abundance. Post hoc comparisons identified six sites with particularly distinct abundance patterns: sites 5, 10, and 17–20 showed significant deviations (p < 0.05) from the overall mean. These results indicate non-random spatial structuring of Agaricales assemblages, with specific microsites supporting markedly higher or lower abundances relative to the study area average.

**Fig. 6:**
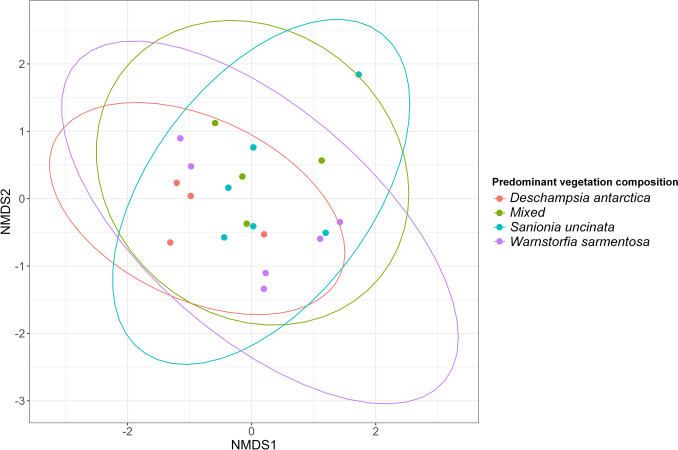
NMDS ordination of Agaricales assemblages based on Bray–Curtis dissimilarity. Points represent sampling sites, colored by dominant vegetation type. The ordination shows partial overlap among vegetation categories and limited segregation of morphospecies composition, based on abundance data

Multivariate patterns in Agaricales assemblage composition were further examined using NMDS ordination based on Bray–Curtis dissimilarity (Fig. 6). The ordination revealed substantial overlap among sites associated with different vegetation categories, indicating limited segregation of assemblages in multivariate space. In agreement with this pattern, PERMANOVA detected no significant differences in morphospecies composition among vegetation types (F = 0.95, R^2^ = 0.15, p = 0.527), indicating that dominant vegetation type alone does not account for a significant proportion of the observed variation in assemblage composition across the study area.

Overall, the results demonstrate pronounced spatial structuring in Agaricales abundance and community composition across the study area, while multivariate analyses indicate only a weak association with dominant vegetation categories. A synthesis of the main statistical outputs, including measures of sampling adequacy, richness estimators, and univariate and multivariate test results, is presented in Table 3, whereas site-level diversity indices are provided in the Supplementary Material.

**Table 3:** Summary of sampling effort, richness estimates, diversity indices, and results of univariate and multivariate statistical analyses for Agaricales assemblages across the study sites

Analysis category	Site/Metric	Statistic/Estimate/*p*-value
Sampling coverage	Total sampling sites	20
Sampling coverage	Total basidiomes	2,566
Richness	Observed morphospecies richness	8
Richness estimator	Chao1	8.0
Richness estimator	Jackknife 1 (Jack1)	8.0
Richness estimator	Jackknife 2 (Jack2)	7.14
Richness estimator	Bootstrap	8.20
Alpha diversity	Shannon–Wiener (H′)	1.97
Alpha diversity	Simpson’s index (1–D)	0.85
Univariate analysis	One-way ANOVA (Agaricales abundance)	0.016
Multivariate analysis	PERMANOVA (vegetation type)	0.527

## Discussion

The documentation of 2,566 basidiomes representing eight Agaricales morphospecies across three genera on an Antarctic island provides new insights into fungal diversity and reproductive activity in polar terrestrial ecosystems. Although species richness was moderate, consistent with expectations for extreme environments such as Antarctica [4, 33], the high number of basidiomes recorded indicates that Agaricales can sustain substantial reproductive output under favorable conditions. Collectively, these findings raise fundamental questions about community assembly, persistence strategies, and the environmental controls governing fungal activity in Antarctica.

When compared with studies conducted in temperate and subtropical ecosystems, our survey yielded basidiome counts that are unexpectedly high given the spatially restricted and climatically harsh Antarctic setting. For example, Araujo et al. [3] documented approximately 1,800 basidiomes in the highly diverse Atlantic Forest of Brazil, Romano et al. [30] reported 681 specimens in *Salix* and *Populus* plantations in Argentina, and Ruiz-Almenara et al. [32] recorded 856 basidiomes during a four-month survey in Mexican forests. Higher absolute abundances have been reported in long-term or large-scale studies, such as Romano et al. [31], who documented 4,072 basidiomata over two years in Argentine Patagonia, and Alem et al. [1], who recorded more than 13,000 sporocarps in Ethiopian Dry Afromontane forests. However, these values reflect substantially longer sampling periods and broader survey areas. Consequently, direct numerical comparisons must be interpreted cautiously due to differences in methodology, spatial coverage, and counting protocols. Rather than emphasizing absolute abundance, our results highlight an underappreciated ecological context in Antarctica, where macrofungal reproductive activity can be locally intense despite moderate overall diversity.

The presence of an active Agaricales community in such a climatically extreme and spatially restricted environment highlights the critical need to investigate fungal diversity and phenology in polar regions. Fungi are highly sensitive to environmental changes, with warming and drought altering ectomycorrhizal networks [15] and global temperatures driving shifts in reproductive phenology [21]. Rising temperatures, changing moisture regimes, and glacial retreat may similarly shape Antarctic fungal communities. Consistent with this, Ma et al. [22] demonstrated that fungal assemblages in the Everest Glacier are strongly structured by altitude and environmental gradients, with climate-driven shifts potentially favoring pathogenic taxa. These insights underscore the importance of long-term, standardized monitoring to elucidate fungal responses to climate variability and their ecological consequences.

Although Antarctic Agaricales remain largely unexplored, evidence from other fungal groups indicates substantial thermal adaptability. Strains of *Penicillium, Fusarium, Rhodotorula*, and additional genera have demonstrated growth at elevated temperatures, including 37 °C [2, 35]. Such findings suggest that Antarctic fungi possess considerable thermal plasticity, with potential implications for pathogenicity and niche expansion under climate warming. Whether Agaricales exhibit similar adaptive capacities remains an open question.

Palfner et al. [26] documented new macrofungal occurrences in Antarctica, expanding the known geographic ranges of several macroscopic species. They suggested that the observed increase in Agaricales diversity and distribution may be linked to regional warming trends along the Antarctic Peninsula. More recently, four new species of *Omphalina* were described from Antarctic environments [6], reinforcing the view that the genus is both more diverse and ecologically relevant in the continent than previously assumed. These findings raise an important question: do these species represent recent introductions facilitated by climate change, or are they long-established populations whose basidiome production was historically constrained by suboptimal thermal conditions during reproductive cycles?

In this context, our findings of eight morphospecies producing basidiomes suggest that these fungi may have persisted as dormant or low-activity mycelial networks for extended periods, with current environmental conditions now enabling reproductive structure formation. Extreme climatic anomalies, such as the March 2022 Antarctic heatwave [9], may temporarily elevate soil temperature and moisture, altering surface microhabitats and potentially promoting fungal development.

Hence, such episodic climatic events, superimposed on long-term warming trends, may act as triggers for reproductive activity in otherwise cryptic fungal communities. Taken together, our results highlight the need to reconsider Antarctica not merely as a marginal environment for Agaricales, but as a dynamic system where subtle climatic fluctuations can unlock hidden fungal potential. This perspective underscores the importance of integrating fungal surveys into broader ecological monitoring programs, as these organisms may act as sensitive sentinels of ecosystem restructuring under ongoing climate change.

## Conclusion

This study provides the first ecological and statistical assessment of Agaricales fungi distribution in Antarctica. These data establish a quantitative baseline for macromycete diversity in the South Shetland Islands and reveal unexpectedly complex community structures in this extreme polar environment, thereby advancing our understanding of Antarctic fungal ecology.

Although based on a single temporal survey conducted in February 2023 and lacking direct environmental measurements such as temperature, humidity, and substrate characteristics, the dataset offers a robust foundation for future comparisons and the development of long-term temporal series. To overcome current limitations, subsequent studies should incorporate repeated sampling across seasons and years, combined with experimental manipulations and molecular approaches, to elucidate the environmental drivers shaping fungal community dynamics under ongoing climate change.

By establishing a reference framework for tracking spatial and temporal variation in Antarctic macromycete assemblages, our findings emphasize the ecological significance of fungi in Antarctic ecosystems—an aspect historically overlooked in conservation strategies. Collectively, this work expands baseline knowledge of polar fungal ecology, underscores the importance of integrating fungal surveys into broader Antarctic management and monitoring programs, and provides essential insights for predicting ecosystem responses to accelerating environmental change.

## Supplementary Information

Below is the link to the electronic supplementary material.

Supplementary file1 (DOCX 16 KB)

## Data Availability

No datasets were generated or analysed during the current study.
