# Supplementary material for: Abundant Development of Agaricales Fungi on Livingston Island, Antarctica: Potential Connections to Climate Change
Source: Curr Microbiol. 2026 Jun 9;83(8):406. doi: 10.1007/s00284-026-04947-6 (PMC13249637; doi:10.1007/s00284-026-04947-6)
Supplement: Supplementary file 1 — Supplementary file1 (DOCX 16 KB) [file 284_2026_4947_MOESM1_ESM.docx]

| Site | Shannon (H′) | Simpson (1−D) |
| --- | --- | --- |
| 1 | 1.32 | 0.70 |
| 2 | 0.25 | 0.11 |
| 3 | 0.0 | 0.0 |
| 4 | 0.68 | 0.49 |
| 5 | 0.0 | 0.0 |
| 6 | 0.54 | 0.35 |
| 7 | 0.09 | 0.03 |
| 8 | 0.58 | 0.32 |
| 9 | 1.04 | 0.63 |
| 10 | 1.18 | 0.66 |
| 11 | 0.69 | 0.49 |
| 12 | 1.16 | 0.64 |
| 13 | 0.46 | 0.28 |
| 14 | 1.47 | 0.70 |
| 15 | 0.32 | 0.14 |
| 16 | 0.24 | 0.12 |
| 17 | 0.0 | 0.0 |
| 18 | 0.0 | 0.0 |
| 19 | 0.0 | 0.0 |
| 20 | 0.0 | 0.0 |

**Supplementary Table:** Shannon and Simpson diversity indices for Agaricales assemblages across sampling sites. Diversity indices were calculated based on basidiome abundance per site. Shannon (H′) reflects both richness and evenness, whereas Simpson (1 − D) emphasizes dominance patterns. Zero values indicate sites where only a single species was recorded.
